# Digital religion and Generation Z: an empirical study in the context of China

**DOI:** 10.3389/fpsyg.2025.1536644

**Published:** 2025-10-02

**Authors:** Zhi Liu, Arsalan Mujahid Ghouri, Jing Wang, Changqing Lin

**Affiliations:** ^1^School of Culture and Communication, Putian University, Fujian Province, Putian, China; ^2^School of Business, London South Bank University, London, United Kingdom

**Keywords:** digital religion, theory of planned behavior, Generation Z, Chinese youth, social media platforms

## Abstract

**Introduction:**

This study investigates the intersection of digital media and religious behavior among Chinese Generation Z, extending the Theory of Planned Behavior (TPB) by incorporating media content innovation and empathic willingness. It explores how platforms like Weibo and TikTok shape religious intentions and behaviors through visual and interactive features.

**Methods:**

A cross-sectional study was conducted with 534 Chinese Generation Z participants. Data were collected via surveys assessing the influence of media content innovation and empathic willingness on digital religious behavior, using the TPB framework. Structural equation modeling analyzed the relationships between these factors and religious intentions.

**Results:**

Findings indicate that media content innovation and empathic willingness significantly enhance digital religious behavior within the TPB framework. Vibrant, emotionally resonant content on social media platforms fosters emotional engagement and active participation, amplifying religious intentions among participants.

**Discussion:**

These results highlight social media’s transformative role in redefining religious practices among Chinese Generation Z. Media content innovation and empathic willingness emerge as critical drivers of engagement. The findings offer insights for religious organizations, policymakers, and social media platforms to foster meaningful cultural and religious interactions. This study underscores the need for tailored digital strategies to promote spiritual engagement in the digital age.

## 1 Introduction

The global proliferation of social media has reshaped how individuals engage with religion and spiritual practices. Individuals are using social media platforms to explore, express, and connect over religious and spiritual content. Therefore, young people increasingly turn to platforms like TikTok, Instagram and Weibo to explore and express their beliefs (Ghouri et al., 2022; Perrin and Anderson, 2019). For Generation Z (born between 1997 and 2012), social media serves as a dynamic “third space” (Prensky, 2001; Szymkowiak et al., 2021). This space bridges traditional and digital realms and fosters unique forms of religious engagement for them (Epafras et al., 2021). This study focuses on the digital religious behavior of Chinese Generation Z who participate in practices such as “worshiping gods and Buddha” on platforms like Weibo and TikTok. The research context is specific to China’s cultural and atheistic landscape. The findings have broader implications for understanding how digital media shapes religious expression across diverse cultural and religious settings worldwide.

Atheism has historically shaped public attitudes toward religion in China. This attitude is changing, and young people engage in digital religious practices that blend traditional rituals with modern media affordances (Li and Chen, 2023). For example, Chinese youth share videos of burning incense to pray for academic success or post emojis and GIFs to express spiritual aspirations (Stahl and Literat, 2023). This behavior is referred to as the “worship craze.” This reflects a pragmatic approach to spirituality where divine intervention is sought for personal goals rather than formal religious affiliation (Wu, 2008; Gall and Guirguis-Younger, 2013; Shen, 2020). Similar patterns are observable around the world: for instance, on one hand, young Catholics in the United States use Instagram to share rosary prayers, on the other hand, Muslim youth in Indonesia create TikTok videos to promote Islamic teachings (Wilkins-Laflamme, 2022a; Subchi et al., 2022). These examples illustrate the universal appeal of digital religion. Social media platforms enable youth to engage with spirituality in many ways with their daily lives, e.g., accessible, interactive, resonant.

The urgency of studying digital religion lies in its role as a transformative force in shaping youth identity (Eaves et al., 2008). Social media platforms amplify emotional and social connections which allow young people to form communities that transcend geographical and cultural boundaries (Evolvi, 2022). For instance, a viral TikTok trend in 2023 saw global Generation Z users participating in virtual pilgrimages where they shared videos of religious sites or rituals. These platforms provided a sense of shared spirituality across cultures and share a peace message. In China, the rise of online temple visits and digital prayer practices highlights how digital platforms bridge online and offline religious experiences. This experience or phenomenon is known as “transmedia” (Scolari, 2015). This behavior provides a lens to examine and extend Theory of Planned Behavior (TPB) framework. Therefore, this study incorporates traditional variables (attitudes, subjective norms, perceived behavioral control) and novel dimensions (media content innovation, image appeal, and empathic willingness) to explore the psychological and social drivers of digital religious behavior.

This research addresses two key questions: (1) What factors of social media content production influence the emergence of religious behavior among young people in China? (2) How can the extended TPB framework, incorporating media content innovation, image appeal, and empathic willingness, explain the religious intentions and behavior of Chinese youth? The findings offer insights into religious organizations, policymakers, and social media platforms globally about Generation Z. Additionally, it illustrates how digital tools can foster spiritual engagement and cultural understanding across diverse contexts. Finally, this study underscores the universal relevance of digital religion in shaping youth identity and community in China in the digital age.

## 2 Theory and concepts

### 2.1 Foundations of the theory of planned behavior and rationale for its extension

TPB is a framework to understand why people do what they do (Ajzen, 1985, 1991). Ajzen (1991) said that wanting to do something depends on how you feel about it, what others think, and how easy it seems to perceive (Ajzen, 1980; Beck and Ajzen, 1991). TPB says if you think something is good, you are more apt to do it (ATB; Ajzen, 1991; Fishbein and Ajzen, 1975). Prior work indicates TPB works to explain how psychological and social events impact what people plan to do (Kim et al., 2016; Mou and Lin, 2015; Taylor and Todd, 1995; Annamdevula et al., 2022; Guggenheim et al., 2020). A lot of studies used TPB to speculate and explain what people do online (Baker and White, 2010; Ali et al., 2018), mostly looking at how religious views change health actions on social media (Reinecke and Oliver, 2017; Seyal and Turner, 2013; Adiyoso and Wilopo., 2021). Studies also found how social media helps people join in religious actions (Solahudin and Fakhruroji, 2020; Bano, 2019; Subchi et al., 2022). These papers often say that attitudes, subjective norms, and perceived behavioral control push online religious actions (Lakhan, 2018). For example, Malik et al. (2023) found these factors really change digital religious plans and actions on places like Facebook. Likewise, Rahman et al. (2015) showed that social norms and feeling in charge are tied to online religious actions for Malaysian Muslims.

In China, theoretical studies on digital religious behavior, particularly among youth, are relatively scarce (e.g., Shen, 2020; Zhang, 2023; Xia, 2023; Wu, 2008). Such research enhances understanding of the factors that influence young people’s willingness to participate in digital religious activities (Shen, 2020; Xia, 2023; Gao et al., 2024). TikTok is the most popular social network platform among Chinese youth, showcasing greater potential than other social networking sites in allowing for easier, faster, and broader improvisation, transmission, and dissemination of information (Chen and Chen, 2018). Understanding the relationship between the factors that how users generate content on social media and digital religious interactions can help explain the psychological and social needs influencing users’ choice of specific media channels and content (Lin, 2006; Malik et al., 2023). However, little is known about how content is generated on social media platforms and why it leads to digital religious intentions and behavior among youth. Moreover, social media has become a critical space for collective online expression by youth (Stahl and Literat, 2023; Literat and Kligler-Vilenchik, 2019).

Individuals frequently share their beliefs or engage with faith-based content online. One key psychological factor driving teens to use social media is its social, messaging, and interactive functions (Qutteina et al., 2022). These features help people understand and share their peer’ feelings (Vossen and Valkenburg, 2016). The current study supports past work, suggesting that creating user-generated content to how well the model explains ATDR (e.g., Miller, 2013; Brubaker and Haigh, 2017; Uzun and Kilis, 2020). Also, being willing to be empathic makes the model powerful at explaining ATDR (e.g., Dovidio et al., 2010; Luchsinger, 2020; Zhao et al., 2016). Still, few studies have put both ideas into the TPB model, and there is lack of research on these variables in social networks (Alemany et al., 2020). This study uses a larger TPB model to explain what people plan to do and actually do when they engage in digital religious actions on social media. The study model has the basic TPB setup, plus things like making social content and being willing to be empathic.

The reason for adding media content innovation and image appeal to the TPB is that media in China has changed a lot. With digital places like Weibo and TikTok, how people use and react to media is very different. Social media sites have new kinds of media content, like news that’s just for you and ways to communicate interactively. These things really change how Generation Z feels and acts. This change fits with what TPB says about how your feelings are a key sign of what you’ll do.

In this context, visually compelling and emotionally resonant content are very important. These visuals make media content better and cause strong feelings. These feelings matter since they change how people feel, what they plan, how they see things, and how they engage and get relevent perception. Incorporating these dimensions into TPB helps us understand how modern media changes what people do. This approach is particularly valuable in the fast-changing world of Chinese digital media.

### 2.2 Social media

Social media has become a powerful tool for individuals seeking visibility by producing content—videos, audio recordings, and more—crafted to attract online audiences (Chang and Zhu, 2011). Its ability to facilitate communication and exchange information, unhindered by physical or temporal constraints, has turned it into an indispensable part of modern life (Abbas et al., 2022). Across generations, social media deeply influences daily interactions and personal experiences (Chu et al., 2016; Alokour and Melhem, 2022). For digital natives, the internet is not just a tool but a woven thread in their lives (Baker and White, 2010; Reinecke et al., 2014), familiar to them since childhood (Supratman, 2018). Additionally, research by Supratman and Wahyudin (2020) highlights how messages promoting religious tolerance, spread via social media, have significantly impacted the Indonesian millennial generation.

In today’s digital age, a range of applications and platforms support religious engagement—daily prayer apps, digital scriptures, and virtual spaces for religious discourse, to name a few. Technological innovation continues to reshape religious practices, particularly among youth in Islamic countries (Nurhasanah, 2024). In Indonesia, adolescents increasingly turn to social media for spiritual guidance (Retpitasari and Oktavia, 2016). They watch short da’wah videos on TikTok (Febriani and Ritonga, 2022) or engage with da’wah posts on Instagram, which foster social connections by offering spaces for interaction among like-minded believers (Purwaningtyas et al., 2024).

Empirical research into mediatization has explored how specific digital media shape the lives of religious communities. Studies examine the influence of media on entire faith-based groups (Nwankwo, 2022), the online dissemination of belief systems (Pavić et al., 2017), and the ability of media to facilitate spiritual meaning-making and interpersonal bonds (Campbell and Zachary, 2022; Lövheim and Hjarvard, 2019). Digital religious media serve many purposes: acquiring information about faith and spirituality (Brubaker and Haigh, 2017), fostering spiritual experiences with transcendent beings (Nduka and McGuire, 2017), and connecting believers within and beyond their communities (Campbell and Zachary, 2022). These connections often take shape through social media platforms or messaging apps (Hodøl, 2021). In Western research circles, considerable focus has also been placed on the intersection of social media and digital religious belief systems (e.g., Diener and Seligman, 2002; Ferriss, 2002; Lim and Putnam, 2010; Stark and Maier, 2008).

Among Generation Z, TikTok stands out as one of the most widely used platforms. According to Kompas.com, 42% of TikTok users fall within the 18–24 age bracket. Like Facebook, Instagram, YouTube, and Twitter, TikTok enables users to interact and share information seamlessly (Lallmahomed et al., 2013). It boasts an array of features—sound effects, voice manipulation, stickers, filters, beauty enhancers, and timers—that make it uniquely engaging (Abbasi et al., 2023). These platforms allow users to upload short videos, typically 3 to 5 min in length, packed with distinctive and vibrant content. Such clips offer an easy and effective way for users to showcase hobbies and everyday activities. TikTok’s characteristic style sets it apart from other platforms and contributes to its immense popularity among Generation Z.

Increasingly, TikTok is being seen as a fertile ground for religious outreach and spiritual expression. Epafras et al. (2021) noted that for Generation Z and young religious minorities, TikTok’s visual culture has become a mainstream channel through which faith is communicated and lived.

### 2.3 Digital religion

“Digital religion” encompasses more than the mere online representation or expression of faith (Zhang, 2019). It involves a dynamic interplay: how digital media and virtual spaces shape religious practices (Ting et al., 2024), and how those practices, in turn, influence the very nature of digital media. This reciprocal relationship impacts both religious expression and belief (Campbell and Evolvi, 2020). Meyer (2006) argued that religion establishes a distance between humans and transcendent entities. In this context, the material presence of online technology acts as a mediator, helping individuals experience a sense of transcendence (Aggarwal et al., 2023). However, Appadurai (2015), building upon Meyer’s ideas, excluded the role of mediation and materiality from his analysis of religious belief.

Still, many scholars insist that digital religion must be understood through the lived experiences of all religious practitioners—human and non-human agents alike (Hoover, 2006). Campbell (2021) emphasized that digital artifacts and technologies like smartphones, tablets, and computers function as material objects capable of embodying religious experience. Similarly, Morgan (2005) noted how the visual culture of social media has become central to digital religious life, enabling sensory engagement with religious imagery. Hutchings (2016) highlighted how a platform’s architecture—its design, aesthetics, coding, and interface—shapes unique practices that contribute to the materiality of digital religion. Campbell and Connelly (2020) further illustrated how individuals use audiovisual media to create religious artifacts digitally, often mirroring their offline spiritual practices. Ultimately, digital technology gives rise to sensory religious practices that unfold through mediated social platforms (Renser and Tiidenberg, 2020; Evolvi, 2022; Douglas, 2005).

### 2.4 Attitude toward digital religion and digital religious intention

Attitude refers to an individual’s positive or negative evaluation of engaging in a specific behavior (Ajzen and Madden, 1986; Fishbein and Ajzen, 1975). For Generation Z, the vivid visual and multisensory experiences offered by TikTok’s video content have played a significant role in shaping their religious understanding and perception of various societal and faith-related phenomena (Epafras et al., 2021). For Generation Z, the vibrant world of interactive and visually captivating media is not just entertainment—it’s a spark that ignites their spiritual curiosity and inspires them to live out their beliefs (Bamualim, 2018; Sari, 2019; Lelono et al., 2020). Their enthusiasm for digital religion shapes how they engage with online spiritual practices (White, 2017). When Generation Z views digital religion favorably—drawn to its accessibility, convenience, and the sense of community it fosters (Pehar et al., 2020)—they are far more likely to weave these practices into their daily lives (Graça and Brandão, 2024). The ability to tap into religious content anytime, anywhere, and connect with a global faith network fuels this openness (Kamarulzaman et al., 2016), making spirituality feel less like a chore and more like a meaningful, personal journey (Campbell, 2012).

Visuals play a starring role in how Generation Z absorbs religious information, with platforms like Weibo and TikTok serving as their primary lens (Epafras et al., 2021). Researchers note that much of what young people share on social media—think selfies at temple visits—often centers on seeking blessings or capturing moments of spiritual significance (McClure, 2016; Murray, 2018). This trend has prompted a broader rethinking of trust and authority in religious spaces, as social media opens new doors for younger audiences to explore faith in fresh, dynamic ways (Zeqiri et al., 2022; Siddiqui and Hamid, 2023). A positive attitude toward digital religion is a key driver, motivating Generation Z to dive into online practices with enthusiasm.

To keep this momentum going, creating supportive and engaging digital spaces is critical (Wang et al., 2024). Religious organizations and leaders have a golden opportunity to meet Generation Z where they are, designing user-friendly platforms that resonate with their preferences and expectations (Luttrell and McGrath, 2021). By addressing concerns—like privacy or authenticity—and highlighting the joys of digital spiritual engagement (Alemany et al., 2020), these groups can build bridges to wider participation (Soylu et al., 2022). Ultimately, fostering a welcoming attitude toward digital religion does not just boost involvement; it breathes new life into faith communities, enriching their vibrancy and growth in our connected world. With this in mind, we propose the following hypothesis:

*H1a:* Attitude toward digital religion has a positive impact on digital religious intention.

### 2.5 Attitude toward digital religion and empathic willingness

Generation Z’s Attitude toward digital religion is deeply rooted in the digital world they have grown up in—a fast-paced, interconnected space that feels as natural as breathing. They’re drawn to platforms that are easy to use, always available, and packed with ways to connect with others. For them, digital religion is not just a handy tool; it’s a lively way to explore faith on their own terms, at a pace that suits their lives (Helland, 2016; Lowe and Lowe, 2018). Whether they are streaming a late-night sermon, joining a virtual prayer group, or diving into interactive spiritual apps, Generation Z finds freedom in accessing religious content whenever the mood strikes (Allal-Chérif, 2022). I remember late nights during my research, scrolling through TikTok to understand how young people engage with spiritual content—those vibrant, bite-sized videos felt like a window into their world, far from the stiff pews of traditional services (Zhou, 2021). To Generation Z, these digital spaces aren’t a weak substitute for in-person worship; they are a dynamic extension of their spiritual journey, adding depth and flexibility to their faith (Li et al., 2023).

Generation Z’s empathic willingness, shaped by a constant stream of diverse voices and global issues on social media (Rawal and Saavedra Torres, 2017). Their passion for social justice and inclusion pulls them toward digital religion that feels compassionate and community-focused. They’re not just after rituals—they want spaces that echo their values of connection, empathy, and mutual support (Bozkurt et al., 2019). This empathetic lens shapes their engagement with digital religion, especially when it aligns with their dream of a more inclusive world.

Religious organizations have a real chance to connect with this mindset. By creating digital content that speaks to Generation Z’s priorities—empathy, authenticity, and social good—they can build online spaces that buzz with life and feel welcoming (Zhou et al., 2022). Platforms that are intuitive and emphasize shared values spark deeper engagement. When the personal and communal perks of digital faith are clear, Generation Z is more likely to weave these practices into their everyday lives, finding meaning where technology and spirituality meet. By tapping into this generation’s heart for inclusion and authenticity, religious communities can grow and thrive in the digital age. Based on these insights, we propose the following hypothesis:

*H1b:* Attitude toward digital religion has a significant positive effect on empathic willingness.

### 2.6 Subjective norms and digital religious intention

Generation Z’s view of digital religion is shaped by their life experiences and the digital landscape they have always known. This generation values convenience, accessibility, and the ability to connect with others through online spaces. Digital religion offers them a flexible environment to explore spiritual beliefs at their own pace and in personalized ways (Helland, 2016; Lowe and Lowe, 2018). The freedom to access religious content anytime, join digital faith communities, and engage with interactive spiritual materials makes digital religion especially appealing (Allal-Chérif, 2022). These platforms aren’t merely substitutes for traditional worship—they enhance spiritual experience by expanding its reach and relevance.

Empathy is a driving force in how Generation Z connects with digital religion. Raised on social media, where diverse voices and stories shape their worldview, they approach faith with a deep sense of compassion and a commitment to inclusivity. They’re drawn to religious content that feels accepting, fosters community, and emphasizes collective care. This empathetic perspective does not just make online spiritual practices appealing—it gives them a deeper purpose, linking Generation Z with others who share their values.

Religious leaders and organizations can tap into this by creating digital content that speaks to Generation Z’s core passions. Focusing on themes like empathy, justice, and community can help faith-based groups build online spaces that feel vibrant and inclusive (Van der Linden, 2011; Kaur, 2020). Platforms that are easy to navigate and reflect the fast-paced, connected lives of young people are more likely to spark active engagement. By clearly highlighting the personal growth and communal benefits of digital faith, leaders can encourage Generation Z to weave these practices into their daily lives (Ghouri et al., 2016; Ghouri et al., 2018). When faith aligns with their values, digital religion does not just take root—it thrives.

Subjective norms, which refer to the social pressure individuals feel to behave a certain way (Ajzen, 1991; Lapinski and Rimal, 2005). Friends, family, and the diverse online communities they interact with carry a lot of weight (Hamilton and White, 2012; Chung and Rimal, 2016). Unlike older generations, who often prioritize in-person worship, Generation Z’s sense of spiritual duty is shaped by the digital age, guided by the beliefs of their social circles, both online and off (Gentina, 2020; La Barbera and Ajzen, 2020; Wilkins-Laflamme, 2022b). Many prefer exploring spirituality through private or virtual channels over traditional gatherings.

Digital platforms have become a cornerstone of Generation Z’s spiritual expression (Epafras et al., 2021). They use social media, apps, and online groups to explore and share their beliefs, adapting traditional practices to fit their on-the-go lifestyles (Reinikainen et al., 2020; Campbell and Connelly, 2020). Many members of Generation Z blend conventional and digital faith practices, creating a hybrid approach that suits their needs (Tirocchi, 2024). For example, some might attend a physical service one day and join an online prayer group the next, staying connected to their faith while embracing digital tools’ convenience (Cooper et al., 2021). For Generation Z, spirituality often ties closely to mental well-being, offering a sense of peace and belonging that supports their emotional and social health. Based on these insights, we propose the following hypothesis:

*H2a:* Subjective norms have a positive impact on digital religious intention.

### 2.7 Subjective norms and empathic willingness

Generation Z’s beliefs are often molded by the people they hold close (Koay et al., 2022). Subjective norms, rooted in the expectations and actions of peers, are profoundly shaped by the digital age (Pandit, 2015). Unlike their parents or grandparents, Generation Z is immersed in online communities and social media, where a swirl of diverse ideas expands their horizons (Duffett, 2017). I recall sifting through X posts during my research, struck by how passionately young people debated justice and inclusion—those raw, unfiltered conversations revealed a generation eager to connect globally (Dhar et al., 2020; Zong and Cheah, 2023). These platforms draw Generation Z into discussions about justice, diversity, and acceptance, nurturing a sense of global citizenship (Roblek et al., 2019). The values they absorb online echo in their actions, guiding how they engage with the world.

Empathy defines much of Generation Z’s identity (Beckmann, 2023). Raised in a torrent of information and varied perspectives, they have honed a sharp awareness of the struggles different groups face. Their empathic willingness shines through in their outspoken support for social movements and their readiness to challenge injustice (Cartabuke et al., 2019). This compassion reaches far beyond their local communities, driven by exposure to global issues through digital media (Tabatabaei et al., 2024). It’s a mindset that ties them to something larger, a sense of shared humanity (Rhodes et al., 2020; Salminen et al., 2021).

This mix of ideological awareness and emotional intelligence spurs Generation Z to act (Confetto et al., 2023). They jump into movements, back charitable causes, and push for change—online and off. Their tech-savvy nature, paired with their empathic willingness, empowers them to shape societal conversations and drive progress across borders (Luttrell and McGrath, 2021). They grasp the interconnectedness of global challenges and respond by working to improve lives (Aggarwal et al., 2024). Building on these insights, we propose the following hypothesis:

*H2b:* Subjective norms have a positive impact on empathic willingness.

### 2.8 Perceived behavioral control and digital religious intention

Generation Z’s Perceived behavioral control plays a pivotal role in shaping their digital religious intention (Ajzen, 1991; Trafimow et al., 2002; Paul et al., 2016). Raised in a world where technology feels like second nature, they navigate digital spaces with ease to explore and express their spirituality. Online platforms offer a convenient, open door to faith, boosting their confidence to join virtual religious practices (Susilawati et al., 2021). I recall pouring over online forums during my research, captivated by how Generation Z seamlessly blended meditation apps and virtual prayer groups into their daily lives—it was like watching faith unfold in real time. This self-assurance lets them weave spiritual practices into their digital routines, whether through mindfulness apps, following spiritual influencers, or attending virtual worship services.

Their digital religious intention is also molded by their social environment. Unlike older generations, Generation Z encounters a rich tapestry of religious perspectives online, nurturing a more open and accepting view of spirituality (Sakdiyakorn et al., 2021). This exposure makes them comfortable diving into diverse faith practices in digital formats (Dongell, 2024). Often, their drive to engage spiritually online comes from a personal quest for growth, connection, and mental well-being. Digital spaces create a welcoming haven for seeking guidance, sharing beliefs, and connecting with others worldwide who share their values (Chukwuma, 2018; Gould, 2015).

This interplay of self-efficacy and online engagement highlights Generation Z’s creative approach to spirituality (White, 2017). They do not cling to traditional forms alone but blend digital and in-person expressions of faith to fit their unique needs. This fusion keeps them spiritually active while embracing technology’s flexibility (Chan and Lee, 2023). For Generation Z, digital religious practices aren’t just stand-ins for older traditions—they enrich spiritual life by offering more personalization and freedom (Priporas et al., 2017). This adaptability shows how deeply technology is woven into their lives, including their spiritual routines. Building on these insights, we propose the following hypothesis:

*H3a:* Perceived behavioural control has a significant positive effect on digital religious intention.

### 2.9 Perceived behavioral control and empathic willingness

Generation Z’s perceived behavioral control—their confidence in their ability to act—is deeply rooted in their digital upbringing (Ang, 2023). Raised with tech at their fingertips, they have mastered digital tools, using them not just for everyday tasks but to fuel activism and connect with their communities (Madden, 2019). This sense of agency in online spaces makes them more likely to dive into issues they care about, like justice, environmental sustainability, or mental health (Vallone et al., 2016; Chae et al., 2024). Their ease with technology gives them a unique edge, letting them amplify causes and rally support through social media with a kind of fearless energy (Jovanovic et al., 2021).

Beyond their tech skills, Generation Z brings a strong dose of empathy to the table (Garai-Fodor et al., 2021). Constant exposure to diverse perspectives and global issues through media has sharpened their awareness of social challenges (Calloway-Thomas, 2010). This empathic willingness drives them to jump into conversations about justice or climate action (Hu et al., 2023). They’re known for their genuine concern for others (Seemiller and Grace, 2017), which pushes them to champion marginalized voices, support meaningful causes (Venkatesh, 2000), and speak up for those who need it most (Luttrell and McGrath, 2021). Their compassion stretches far, reflecting the connected world they have grown up in (Leong et al., 2024).

This combination of digital confidence and empathic willingness makes Generation Z a powerful force for change (Raman et al., 2024). This generation does not just understand the challenges around them—they believe they can help solve them. Their blend of technological savvy and emotional intelligence equips them to drive change. They influence public discourse, inspire collective action, and strive to build a better future (Luttrell and McGrath, 2021). By harnessing digital tools and embracing empathy, Generation Z is poised to be a transformative force in today’s world (Tohit and Haque, 2024). Building on these insights, we propose the following hypothesis:

*H3b:* Perceived behavioural control has a significant positive effect on empathic willingness.

### 2.10 Media content innovation and image appeal and digital religious intention

Generation Z’s fondness for authentic, relatable content has reshaped how religious messages come to life online (Belvin, 2024). Their craving for real, easy-to-connect-with material has pushed religious organizations and influencers to rethink their approach to spiritual teachings. Increasingly, they are turning to platforms like TikTok, Instagram, and YouTube to share Media content innovation that clicks with Generation Z’s digital religious intention (Myers et al., 2023). These platforms make it easy to craft short, engaging videos and visually striking posts that grab younger audiences’ attention, making faith feel more approachable and relevant (Picazo-Vela et al., 2010).

The image appeal of online content is a big deal for Generation Z’s digital religious engagement (Cam et al., 2024). They’re drawn to posts and videos that look good and feel creative, with eye-catching designs that stand out in their feeds. Religious groups have caught on, weaving dynamic graphics and fresh layouts into their strategies to boost the image appeal of their messages (Wibowo et al., 2020). This approach does not just make content more attractive—it simplifies complex spiritual ideas. Through visual storytelling, themes like diversity, inclusion, and fairness, which Generation Z holds dear, shine through, making the content feel more meaningful and engaging (White, 2017).

Generation Z’s shifting media habits have also sparked new ways to connect with faith online (Podara et al., 2021). Mobile-friendly formats, like short-form videos or disappearing stories, let them soak up religious content on the go (Aggarwal et al., 2024). Streaming platforms and on-demand services bring sermons, spiritual talks, and guidance right to their fingertips. Religious organizations are even tapping into advanced tech, like AI-driven tools and content customization, to tailor messages to Generation Z’s unique interests and spiritual needs. These innovations create personalized, immersive experiences that make digital religious intention more impactful and relevant for young users (Hines, 2024). Building on these insights, we propose the following hypothesis:

*H4a:* Media content innovation and image appeal has a significant positive effect on digital religious intention.

### 2.11 Media content innovation and image appeal and empathic willingness

Generation Z’s engagement is defined by their empathic willingness to connect with others’ experiences. Their hunger for authentic, relatable content has reshaped today’s media landscape, with empathy taking center stage (Tirocchi, 2024). Social media is brimming with personal stories, calls for justice, and community-driven posts that foster a deep sense of connection among users (Fiffer, 2022). This considerate mindset keeps pushing media to be more inclusive and compassionate, reflecting Generation Z’s desire for content that feels real and kind.

The image appeal of Media content innovation plays a big part in enhancing Generation Z’s empathy. Prominent, well-crafted visuals grab their attention and spark emotional reactions (Marmarosh et al., 2020). When done right, these visuals do not just look good—they make messages hit harder and feel more meaningful. Through powerful images and visual storytelling, social challenges, personal struggles, and moments of hope come to life in ways that resonate deeply with viewers (White, 2017). Through the use of vibrant colors, creative graphics, and modern design, content creators craft media that appeals to Generation Z’s aesthetic preferences while deepening their emotional understanding (Zaiddi and Ismail, 2023).

New methods of presenting content continue to build empathy within Generation Z. Short videos, live streams, and interactive formats enable quick emotional engagement (Sun and Yin, 2022). Streaming and on-demand platforms encourage viewers to dive into stories and complex topics, offering opportunities to explore different perspectives (Datta, 2023). Media platforms now employ advanced technologies and AI to personalize content delivery. These innovations help align media with Generation Z’s values and day-to-day experiences. This tailored approach has the potential to strengthen empathy and foster a more connected, compassionate online community. Based on this discussion, we propose the following hypothesis:

*H4b:* Media content innovation and image appeal has a significant positive effect on empathic willingness.

### 2.12 Digital religious intention and digital religious behavior

Generation Z’s ease with technology and social media has reshaped how they connect with religious content and communities. They do not just use online platforms for fun—they dive into them to explore and share their beliefs, shaping their Digital religious intention (Epafras et al., 2021; Hayes, 2021). Whether following spiritual leaders, joining virtual worship, or seeking content that mirrors their values, Generation Z makes faith feel personal and within reach (Battista, 2024). This move to digital spaces meets their diverse spiritual needs, letting them engage with religious intention in ways that feel true to them (Johnston, 2018).

The visual and interactive nature of digital media is a game-changer for Generation Z’s digital religious behavior (White, 2017). Striking images, lively videos, and interactive posts make spiritual teachings pop, turning complex ideas into something easier to grasp (Maina, 2023). For example, short videos or live streams create real-time connections, helping users feel part of a broader spiritual community (He et al., 2023). This kind of engagement gets Generation Z talking about faith, sharing spiritual moments, and supporting each other’s religious behavior (Bauman et al., 2014). Visually appealing content also breaks down tricky religious concepts, deepening their understanding and commitment to faith (Male, 2019).

New trends in digital media are also pushing Generation Z to explore spirituality in fresh ways (Rafsanjani et al., 2024). With religious resources always at their fingertips, they can watch sermons, dig into their faith (McKay and Whitehouse, 2015), or find guidance whenever it suits them—even with packed schedules. AI-powered algorithms serve up tailored content that aligns with their interests and spiritual goals, keeping their Digital religious intention alive and vibrant (Appleby et al., 2010). This personalized approach strengthens their sense of religious identity and fuels their religious behavior in meaningful ways (Soukup et al., 2019). By blending online spirituality with cutting-edge media, Generation Z is redefining how faith is lived and expressed today. Building on these insights, we propose the following hypothesis:

*H5a:* Digital religious intention has a significant positive effect on digital religious behavior.

### 2.13 Empathic willingness and digital religious behavior

Generation Z’s empathic willingness significantly influences their digital religious behavior, creating a unique intersection between emotional engagement and spiritual practice. Their capacity for empathy leads them to seek out and interact with religious material that resonates emotionally (Missier, 2022). Platforms like TikTok, Instagram, and YouTube are rich with stories of faith, spiritual journeys, and personal struggles, which foster emotional bonds and build a sense of community—an essential aspect of Generation Z’s digital religious engagement (Peterson, 2024; Hassan and Kodwani, 2020; Missier, 2022).

The visual power of digital media enhances these connections. Appealing visuals and interactive content make religious teachings more relatable and impactful. Live streams of services and QandA sessions, for example, promote real-time interaction, making religious content more accessible and encouraging active participation (Galen, 2012). Generation Z cultivates digital communities rooted in empathy by sharing personal experiences and supporting one another’s spiritual paths (White, 2017).

Advancements in digital media have introduced new expressions of religious behavior driven by empathy. On-demand spiritual content enables Generation Z to access guidance when it suits their lives (Tran and Nguyen, 2021). Algorithms further personalize these experiences by offering content tailored to individual interests and spiritual needs. This personalization fosters deeper involvement and strengthens faith practices. By weaving empathy into their spiritual experiences, Generation Z is reshaping how faith is lived online, fostering more connected and emotionally engaged digital religious communities (Bingaman, 2020). Based on the discussion, we propose the following hypothesis:

*H5b:* Empathic willingness has a significant positive effect on digital religious behavior.

### 2.14 Research framework

To the best of the researchers’ knowledge, the limited empirical studies on the religious behavior of Chinese youth have primarily focused either on offline religious activities (Feuchtwang, 2003; Chau, 2008; Lin, 2006) or online religious behavior (Shen, 2020; Xia, 2023). Few have explored both dimensions simultaneously or considered other common forms of religious practice. Moreover, prior research has highlighted that the type of religious behavior being assessed plays a key role in influencing the frequency of reported attitudes and practices (Campbell, 2017). This suggests that the evaluation method itself can shape respondents’ perceptions and willingness to disclose their religious activities.

This study investigates three distinct forms of religious behavior shaped by digital engagement: online prayer, offline practices inspired by online religious content, and blended activities occurring both online and offline simultaneously. These represent common and recognizable modalities through which Chinese youth experience and express their religiosity in the digital age. In doing so, this study extends the Theory of Planned Behavior (TPB) framework to account for digital religious behavior among Chinese youth. Specifically, it seeks to examine the direct influence of social identity and empathic needs, and how these variables shape digital religious practices.

## 3 Methodology

### 3.1 Sample

This study employed a purposive sampling approach to target Chinese Generation Z social media users, specifically those active on Weibo and TikTok, as these platforms are among the most popular among this demographic in China. A questionnaire survey was distributed to 30,000 qualified internet users (15,000 Weibo users and 15,000 TikTok users) using the “@” function on Weibo and the private messaging function on TikTok. Participants were invited to complete the survey if they met the inclusion criteria: being born between 1997 and 2012 (Generation Z) and actively engaging with religious or spiritual content on these platforms. To incentivize participation, respondents were offered 30 RMB (approximately 4.80 USD) as compensation. A total of 576 users provided valid responses, and after excluding incomplete or invalid submissions (e.g., responses with missing data or inconsistent answers), the final sample consisted of 534 valid questionnaires (223 males, 311 females), yielding an effective response rate of 92.53%. According to Ding et al. (1995), a sample size of 100 to 150 is sufficient for structural equation modeling (SEM) analysis. The sample size of 534 exceeds this threshold, ensuring robust statistical power for the analyses conducted.

To address potential selection bias, particularly self-selection effects, several measures were implemented. Self-selection bias is a concern in online surveys, as individuals who choose to participate may differ systematically from those who do not, potentially skewing results (Bethlehem, 2010). To reduce self-selection, we recruited participants from two big social media sites, aiming for a varied group. We used specific ways to reach people on each platform (Weibo’s @ and TikTok’s private messages). This let us get participants who are interested in religious content to different degrees. This approach lowered the possibility that only very interested or religious people would take part. Plus, we offered 30 RMB to pull in a larger variety of users, even those who usually skip surveys.

To check for self-selection issues, we looked at things like age, gender, and education in our group and compared them with stats on Chinese Generation Z social media users from studies like Stahl and Literat (2023). Our group’s numbers (41.76% male, 58.24% female, 73.97% undergraduate, 26.03% postgraduate) were like the bigger trends, which hints at representation. Still, some issues exist. People without Weibo or TikTok and people who are uninterested in religion may be underrepresented. Future work could use stratified sampling or quota sampling to make the group even more representative.

### 3.2 Measurement

Demographic information such as age, gender, and educational level was collected from participants. The latent variables included in the model for this study are social norms, digital religious attitudes, perceived behavioral control, image appeal and content innovation, digital religious intentions, empathic willingness, and digital religious behavior, generated by corresponding observed variables. All items were measured using a five-point Likert scale. Below is a brief introduction to the scale:

**Attitudes Toward Online Religion**: Measured using 6 items (e.g., “Participating in digital religious blessing activities is helpful for me”), ranging from 1 (strongly disagree) to 5 (strongly agree) to assess respondents’ attitudes toward digital religion.
**Subjective Norms**: Measured using 6 items (e.g., “My family or friends frequently recommend digital religion to me”), ranging from 1 (never) to 5 (always) to assess the frequency of various forms of digital religious behavior reported by respondents in close relationships.
**Perceived Behavioral Control**: Measured using 5 items (e.g., “Seeking deities and offering prayers” on social media is easy for me) to measure the likelihood of engaging in digital religious activities.
**Media Content Innovation and Image Appeal:** Measured using 4 items, including 2 items for content innovation (e.g., “I enjoy watching religious audiovisual content or materials with interesting perspectives”) and 2 items for image appeal (e.g., “The vivid and novel representations of deities on social media attract me”), with all items ranging from 1 (strongly disagree) to 5 (strongly agree).
**Digital Religious Intention**: Measured using 5 items (e.g., “I am considering participating in ‘worshiping gods and Buddha’ activities through social media”), ranging from 1 (very unlikely) to 5 (very likely) to assess the likelihood of respondents’ digital religious intentions.
**Empathic Willingness**: Measured using 5 items (e.g., “Sharing religious blessing content with others on social media makes me feel a sense of belonging”), ranging from 1 (strongly disagree) to 5 (strongly agree).
**Digital Religious Behavior**: Measured using 5 items (e.g., “I engage in religious blessing activities on social media” and “I frequently search for and participate in offline religious blessing activities related to what I find on social media”), ranging from 1 (never) to 5 (always), to evaluate the frequency of various forms of digital religious behaviors reported by respondents.

To ensure the clarity and accessibility of the questionnaire, especially for participants with weaker English proficiency, a standard back-translation procedure was followed (Brislin, 1986). The questionnaire was translated from English to Chinese by a bilingual researcher, then back-translated to English by a second independent translator to verify accuracy. A pilot test was conducted with 80 Chinese Generation Z students to confirm item comprehensibility and relevance. No issues were identified, and the questionnaire was deemed suitable for the main study.

To mitigate common method bias (CMB), which can arise when data are collected using a single method (e.g., self-reported questionnaires), several procedural and statistical strategies were employed, following recommendations by Podsakoff et al. (2003). Procedurally, the questionnaire was designed to ensure respondent anonymity, reducing social desirability bias. Clear instructions emphasized that there were no right or wrong answers, encouraging honest responses. Items were randomized to avoid order effects, and different response scales were used for different constructs (e.g., frequency-based for subjective norms, likelihood-based for digital religious intention) to minimize response pattern biases. Statistically, Harman’s single-factor test was conducted to assess CMB. The results showed that the first factor accounted for only 32.4% of the total variance, well below the 50% threshold, suggesting that CMB was not a significant concern. Additionally, the high Cronbach’s alpha and composite reliability (CR) values (all > 0.8) and adequate average variance extracted (AVE) values (> 0.5) for all constructs (see Table 1) further indicate that the measurement model was robust and not unduly influenced by CMB.

**Table 1:** Demographics of respondents (*N* = 534).

	M	Percent (%)
Gender		
Male	223	41.76
Female	311	58.24
Age		
18–19	162	30.33
20–22	137	25.65
22–25	235	44.02
Educational background		
Undergraduate	395	73.97
Postgraduate	139	26.03
Religion		
Christianity	0	0
Buddhism	0	0
others	0	0

## 4 Data results

### 4.1 Demographic results

The demographic characteristics of the participants are presented in Table 1. In the sample, about 41.76% of the participants were males, and the others were females. A majority of participants had undergraduate education, with 73.97%, and a share of 26.03% had postgraduate education. All participants reported no religious affiliation.

### 4.2 CFA test results

The researchers evaluated the reliability and validity of each construct. As shown in Table 2, the Cronbach’s alpha and composite reliability (CR) for all items exceeded 0.8, and both factor loadings and average variance extracted (AVE) were greater than 0.5. This indicates that the items demonstrate good reliability and convergent validity (Taber, 2018). Next, we will examine the fit indices of the proposed structural model to validate the research hypotheses.

**Table 2:** Factor loadings, CR, and AVE of items.

Constructs	Factor loading	CR	AVE
Attitudes toward digital religion (Molinillo et al., 2018)			
AT1: The method of “worshiping gods and Buddha” on social media is useful for me.	0.713	0.871	0.53
AT2: The method of “worshiping gods and Buddha” on social media is convenient for me.	0.724		
AT3: “Worshiping gods and Buddha” on social media is necessary for me.	0.753		
AT4: The content shared on social media related to “worshiping gods and Buddha” is helpful for me.	0.719		
AT5: All “worshiping gods and Buddha” activities should be taken seriously.	0.740		
AT6: “Worshiping gods and Buddha” helps society to alleviate stress.	0.719		
Media content innovation and image appeal			
1. The vivid and novel representations of deities on social media attract me.	0.769	0.825	0.542
2. The blessings and spiritual solace provided by the deities attract me.	0.720		
3. I enjoy watching religious audiovisual works or content with interesting perspectives.	0.703		
4. I enjoy watching religious audiovisual works or content with unexpected perspectives.	0.751		
Social norms (Griffin et al., 1987)			
SN1: The frequency with which my family recommends “worshiping gods and Buddha” influences me.	0.759	0.898	0.595
SN2: The frequency with which my friends recommend “worshiping gods and Buddha” influences me.	0.767		
SN3: The frequency with which some groups I belong to share “worshiping gods and Buddha” via social media influences me.	0.821		
SN4: The frequency with which my family shares “worshiping gods and Buddha” content via social media influences me.	0.709		
SN5: The frequency with which my friends share “worshiping gods and Buddha” content via social media influences me.	0.810		
SN6: The frequency with which some groups I belong to share “worshiping gods and Buddha” content via social media influences me.	0.756		
Perceived behavioral control (Wang and Wong, 2021)			
PBC1: “Worshiping gods and Buddha” on social media is easy for me.	0.726	0.881	0.598
PBC2: “Worshiping gods and Buddha” offline is easy for me.	0.793		
PBC3: Accessing “worshiping gods and Buddha” content on social media is easy for me.	0.791		
PBC4: Whether I engage in “worshiping gods and Buddha” is entirely up to me.	0.783		
PBC5: I have the resources, time, and opportunities to participate in “worshiping gods and Buddha” activities online/offline.	0.771		
Digital religious intention (Hwang, 2018)			
IT1: I am considering participating in “worshiping gods and Buddha” activities through social media.	0.719	0.849	0.529
IT2: I am considering attending offline “worshiping gods and Buddha” activities when I learn about them through social media.	0.694		
IT3: I am considering sharing “worshiping gods and Buddha” activities via social media with others.	0.724		
IT4: I would consider recommending others to participate in offline “worshiping gods and Buddha” activities.	0.779		
IT5: I would participate in “worshiping gods and Buddha” activities if given the opportunity.	0.719		
Empathic willingness (Alhabash et al., 2013)			
EC1: Participating in “worshiping gods and Buddha” activities when I am upset or stressed helps me feel emotionally relaxed.	0.867	0.906	0.659
EC2: I feel happy when participating in “worshiping gods and Buddha” activities.	0.761		
EC3: Sharing or interacting with others regarding “worshiping gods and Buddha” on social media fulfills my emotional needs.	0.792		
EC4: Participating in “worshiping gods and Buddha” activities positively affects my sense of empathy.	0.839		
EC5: I feel happy when I know that my friends or acquaintances are participating in “worshiping gods and Buddha” activities on social media.	0.795		
Digital religious behavior (Vilchinsky and Kravetz, 2005)			
BE1: The frequency of participating in online “worshiping gods and Buddha” activities via social media.	0.728	0.846	0.524
BE2: The frequency of attending offline “worshiping gods and Buddha” activities after learning about them via social media.	0.798		
BE3: The frequency of sharing “worshiping gods and Buddha” activities via social media.	0.678		
BE4: The frequency of participating in online “worshiping gods and Buddha” activities with others.	0.746		
BE5: The frequency of attending offline “worshiping gods and Buddha” activities with others.	0.662		

The correlation coefficients of the questionnaire items are presented in Table 3, where the square roots of all AVEs are larger than the correlation coefficients between constructs, satisfying the discriminant validity requirement.

**Table 3:** Correlations between variables.

Constructs	1	2	3	4	5	6	7
1 ATDR	0.728						
2 MCIandIA	0.459***	0.736					
3 SN	0.414***	0.649***	0.771				
4 PBC	0.453***	0.635***	0.66***	0.773			
5 DRI	0.455***	0.596***	0.547***	0.553***	0.727		
6 EW	0.368***	0.481***	0.505***	0.482***	0.406***	0.812	
7 DRB	0.561***	0.563***	0.549***	0.66***	0.572***	0.601***	0.724

The square root of AVE values is noted on the diagonal in bold; *p ≤ 0.05; **p ≤ 0.01; ***p ≤ 0.001.

### 4.3 Structural model testing

The structural model fit evaluation requires that the model fit indices meet the established criteria. Table 4 presents the test results and fit indices for the proposed research model. The results in Table 4 clearly indicate that the fit indices for research model exhibit a good fit. Therefore, the structural model can be used for further hypothesis testing.

**Table 4:** Fit statistics for research model.

Fit index	Satisfied	Model
x^2^		787.983
df		316
RMSEA	0.00 ≤ RMSEA ≤ 0.08	0.053
GFI	0.85 ≤ GFI ≤ 1.00	0.888
CFI	0.85 ≤ CFI ≤ 1.00	0.942
NFI	0.85 ≤ NFI ≤ 1.00	0.908
TLI	0.85 ≤ TFI ≤ 1.00	0.936
x^2^ /df	0.00 ≤ x^2^ /df ≤ 5.00	2.494

### 4.4 Hypothesis testing

The researcher adopted AMOS software to perform path analysis. Table 5 display the standardized regression coefficients for the research model analyzed in this study to test the significant relationships between the constructs and examine whether the collected data support the hypotheses (e.g., Ghouri et al., 2022; Ghouri et al., 2020).

**Table 5:** Standardized path coefficients to testing the causal effects of the constructs for model.

Hypothesis	Construct	Path	Construct	Estimate	SE	*t*-value	Result
H1a	ATOR	→	DRI	0.175	0.076	3.707**	Support
H1b	ATOR	→	EW	0.136	0.061	2.584*	Support
H2a	SN	→	DRI	0.171	0.072	2.823*	Support
H2b	SN	→	EW	0.239	0.058	3.463**	Support
H3a	PBC	→	DRI	0.167	0.079	2.692*	Support
H3b	PBC	→	EW	0.152	0.063	2.187*	Support
H4a	MCIandIA	→	DRI	0.301	0.08	4.668**	Support
H4b	MCIandIA	→	EW	0.171	0.063	2.385*	Support
H5a	DRI	→	DRB	0.238	0.038	4.997**	Support
H5b	EW	→	DRB	0.331	0.056	6.665**	Support

**p ≤ 0.05; *p ≤ 0.01.

As seen in Table 5, all constructs significantly contribute to endogenous constructs. In this model we added a new variable in TRB, i.e., MCIandIA to find the best fit for explaining the DRB of youth on social media platforms. As shown in Table 5, ATOR, SN, PBC and MCIandIA are significantly related to both DRI and EW, supporting the relevant hypotheses. Specifically, ATDR (*β* = 0.136, *p* < 0.05), SN (*β* = 0.239, *p* < 0.001), PBC (*β* = 0.152, *p* < 0.05), and MCIandIA (*β* = 0.171, *p* < 0.05) are significantly related to EW, collectively explaining 38.83% of the variance. Meanwhile, ATOR (*β* = 0.175, *p* < 0.001), SN (*β* = 0.171, *p* < 0.01), PBC (*β* = 0.167, *p* < 0.01), and MCIandIA (*β* = 0.301, *p* < 0.001) are significantly related to DRI, collectively explaining 35.883% of the variance. Additionally, DRI (*β* = 0.238, *p* < 0.005) and EW (*β* = 0.331, *p* < 0.005) are significantly related to DRB, collectively explaining 40.398% of the variance. Thus, all hypotheses were supported.

## 5 Discussion

This study utilized an extended TPB framework to investigate the psychological and social determinants of digital religious intention DRI and DRB among Chinese Generation Z. The findings confirm that attitudes toward digital religion (ATDR), subjective norms (SN), perceived behavior control (PBC), and media content innovation and image appeal (MCIandIA) significantly predict digital religious intention (DRI) and empathic willingness (EW), which in turn influence digital religious behavior (DRB). These results align with prior research (e.g., Malik et al., 2023; Rahman et al., 2015) but extend the TPB by demonstrating the critical role of MCIandIA and EW in shaping digital religious practices, particularly in a context like China, where formal religious affiliation is less common. SN emerged as a significant predictor of DRI and DRB. This finding aligns with earlier studies (Zhao et al., 2016; Idris et al., 2016). The rise of atheistic tendencies in society may explain why SN—the perceived views of social group members—do not always direct DRB (Xia, 2023). Yet, social media’s visual power changes the game. Videos and images, packed with emotional and visual contents, bring religious content to life. The contents capturing sacred rituals, striking images of sacrificial acts, or vivid depictions of spiritual teachings—these elements speak directly to the heart, far beyond what plain text can do. During this research, we analyzed Weibo posts, found that how a single, vibrant image of a candlelit vigil could spark comments filled with spiritual reflections. This kind of visual storytelling does not just stick in the mind; it stirs emotions, deepens impact, and makes content more memorable.

When this visually rich content spreads across social media, it amplifies the influence of SN. Users feel a stronger pull from social expectations, nudging them toward forming DRI and engaging in DRB. As Hutchings (2016) noted, social media’s visual materiality—its design, aesthetics, coding, and interfaces—creates fertile ground for DRI and DRB. The way videos and images are crafted, with their knack for emotional expressiveness, personalized recommendations, and interactive features, boosts SN’s role in shaping DRI. This not only enriches our understanding of social media’s impact on digital religion but also offers practical insights for religious organizations and individuals looking to share faith online.

PBC strongly shapes DRB, a finding echoed in prior studies (Fu and Cook, 2020; Gangneux, 2019; Stahl and Literat, 2023; Zeng et al., 2021). Social media’s unique features amplify users’ confidence in participating in religious activities, driving their digital DRI and DRB. TikTok findings provided how young users shared profound virtual prayers that sparked thousands of supportive comments—it shows faith was alive in those fleeting videos. PBC directly predicts DRB, as seen in earlier work (Hendy and Montargot, 2019). In a world where youth face relentless pressure to succeed, social media’s interactive tools—like comments, likes, and shares—offer instant feedback and emotional support. This community encouragement boosts users’ confidence, reinforcing their sense of ability to engage in religious practices and positively shaping their DRI and DRB.

MCIandIA also play a key role in driving DRI, aligning with prior research (Fu and Cook, 2020; Gangneux, 2019; Stahl and Literat, 2023; Zeng et al., 2021). Social media’s creative formats and engaging visuals reshape how audiences view traditional religion, fostering stronger DRI and DRB. These platforms soften the rigid structures of conventional faith, making religion feel more open and approachable to young people (McClure, 2016; Murray, 2018). Innovations like augmented reality (AR), virtual reality (VR), and interactive videos let users join remote pilgrimages or rituals virtually (Döveling and Konijn, 2022). These dynamic formats do not just draw people in—they spark curiosity and deepen engagement, encouraging active participation in religious activities.

**Figure 1:**
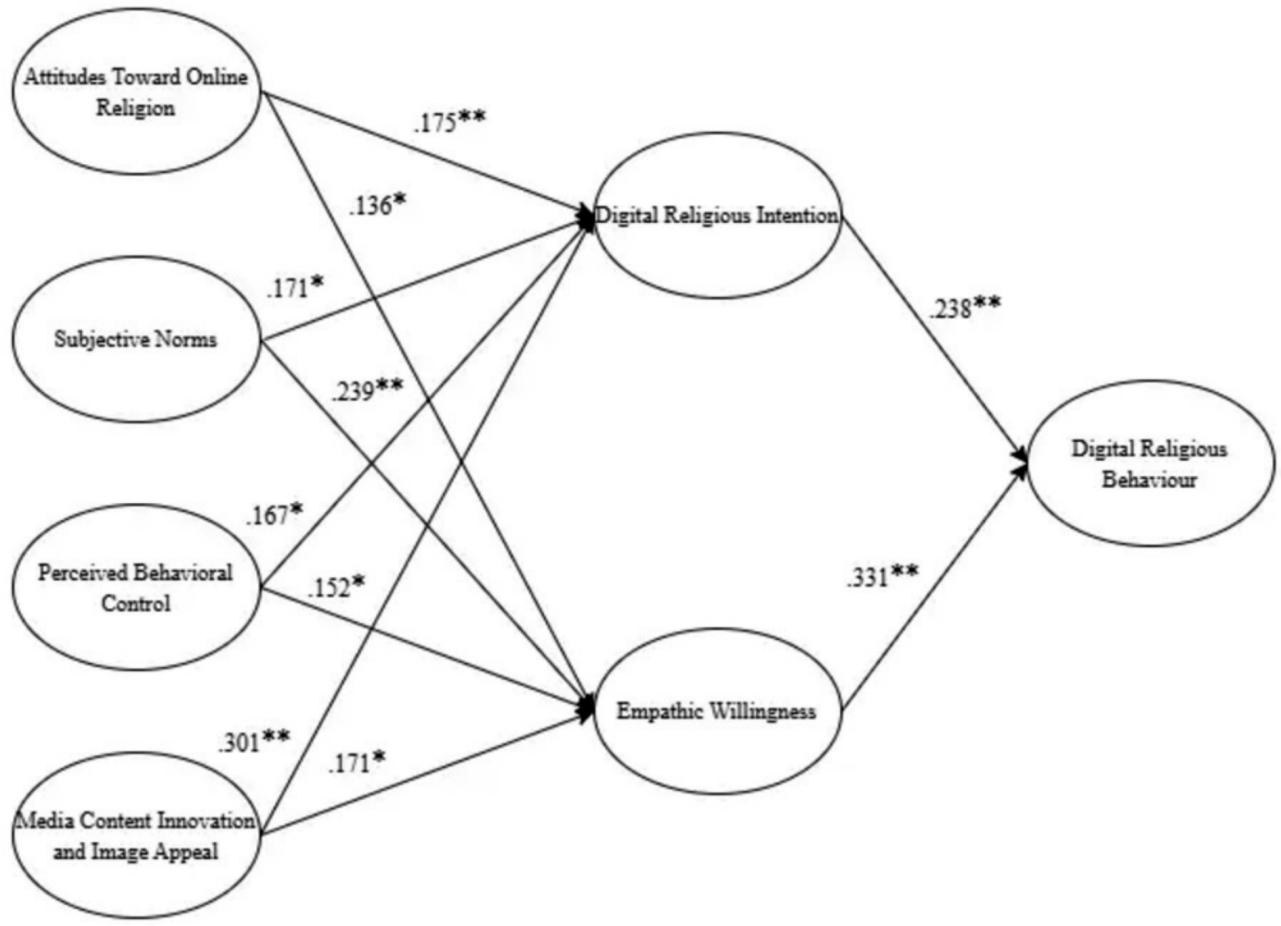
Results of structural equation research model.

ATOR significantly influences DRI and DRB, consistent with past studies (Luchsinger, 2020; McKeever, 2015; Šisler, 2017). Among Chinese youth, ATOR is shaped by cultural roots, personal experiences, and how religious content is presented online. When they encounter faith-based posts on social media, their attitudes begin to take shape, often growing more positive with content that feels relatable and meaningful. Social media blends traditional religious content with modern expressions, like digital rituals or online faith communities, making religion more appealing to young users (Wahid, 2024). This accessibility draws them in, fostering deeper religious engagement (Figure 1).

The power of MCIandIA shines through in visually captivating content that drives spiritual connection. On TikTok, Chinese youth share vibrant animations of deities or virtual incense-burning rituals, sparking thousands of likes and comments while building a sense of shared faith (Stahl and Literat, 2023; Eloundou-Enyegue, 2025). These findings highlight social media’s role in modern religious practices and offer practical strategies for religious organizations to engage young people through innovative, visually rich content. This digital behavior aligns with global trends, e.g., Christian influencers in the United States using Instagram Reels to share Bible verses with dynamic graphics or Hindu youth in India posting devotional songs on YouTube (Myers et al., 2023). These real-world examples illustrate how social media’s visual and interactive features transcend cultural boundaries and making religious content more accessible and appealing to young audiences worldwide.

The study also identified EW as a significant variable in Chinese youth’s participation in DRB, particularly in enhancing the effect of media content innovation and image appeal on DRB. The role of EW as a mediator highlights the emotional and social dimensions of digital religion. Young people in China facing academic and career pressures often turn to online religious content for emotional solace (Ye and Liu, 2022). They are sharing posts about praying for success in university entrance exams (Xia, 2023). Similarly, Muslim youth in the United Kingdom joining virtual Ramadan prayer sessions on Zoom to connect with peers or Buddhist youth in Thailand using LINE to share mindfulness meditation videos (Zaid et al., 2022). These practices demonstrate how EW fosters a sense of belonging and emotional support to specific community as they sustained engagement with digital religious content across diverse cultural settings.

### 5.1 Implications for theory and practice

#### 5.1.1 Theoretical implications

This research enriches the TPB by including MCIandIA and EW. It is offering a detailed look at digital religious actions in a country like China, which is not very religious. The results show that these things aren’t just for Chinese young people; they also match worldwide trends in digital religion. For example, the focus on visual stuff is related to research on Instagram and YouTube’s digital religious actions in Western countries. These digital broadcasts make spiritual involvement stronger through visuals and interaction (Campbell and Connelly, 2020). The important role of EW gives a fresh way to study how feelings drive religious actions, which can be used in different religious and cultural places. Future studies could look at these things in other areas, like Islamic or Christian groups, to see if the expanded TPB model can be used in many places.

#### 5.1.2 Practical implications

The findings offer actionable insights for religious organizations and social media platforms worldwide. Religious organizations in China can leverage platforms like Weibo and TikTok to create visually compelling religious content. These contents could offer a virtual temple tours or animated deity stories to engage youth of China or same belief group. Globally, similar strategies can be applied for Christianity. As Chistian churches could develop TikTok campaigns featuring short sermons with vibrant visuals. Islamic entities could use Instagram Stories to share Hadith with interactive polls. Policymakers can draw on these insights to promote positive digital religious engagement with fostering cultural understanding and social cohesion. Supporting interfaith social media initiatives could bridge divides in pluralistic societies. One of the pieces of evidence of such projects is the #InterfaithHarmony campaign on Twitter. This campaign encourages youth to share messages of religious tolerance.

While addressing the universal appeal of digital religion and providing concrete evidence, the present study highlights its relevance to diverse audiences. Social media’s ability to connect young people across cultures underscores the need for inclusive engaging and empathetic digital religious practices that resonate globally.

## 6 Limitations

This study on digital religious behavior is conducted within the context of an atheist state, thus presenting certain limitations regarding the generalizability of its findings across diverse cultural and religious backgrounds. First, the sample focuses specifically on Generation Z youth in mainland China, whose religious behavior is profoundly shaped by the state’s official atheism (Wang et al., 2024) and exhibits distinctly pragmatic characteristics (Shen, 2020). This specific socio-cultural context means the findings may not be directly generalizable to socio-cultural environments characterized by deep-rooted institutionalized religious traditions, different state-religion relationships, or greater religious freedom. Second, although demographic variables such as participants’ gender and age were collected, the study did not further analyze potential differences in digital religious behavior across gender groups. Furthermore, all participants possessed a higher education background and explicitly reported no specific religious affiliation. While this reflects a significant characteristic of Chinese youth, it limits the applicability of the findings to youth populations with different educational levels, those holding specific religious beliefs, or those situated in environments with greater religious diversity. Third, the research methodology relied primarily on a cross-sectional survey. While effective for capturing behavioral intentions and actual behavior at a specific point in time, it is unable to deeply track the dynamic evolution of digital religious behavior over time or the complex underlying socio-psychological mechanisms. Additionally, the measurement of social media content innovation and image appeal did not fully encompass all dimensions, representations, and manifestations within this domain. Future research could enhance generalizability by incorporating more religiously diverse samples across broader cultural contexts at the individual level. Employing longitudinal or mixed-methods approaches and deepening the multidimensional measurement of social media content features would help validate the extended theoretical model proposed in this study among wider populations, thereby advancing the understanding of Generation Z’s digital religious behavior globally.

## 7 Conclusion

This study collected data from two social media platforms (Weibo and TikTok) to evaluate the extended TBP model. The fit indices indicate that the research model proposed in this study are acceptable and achieve a good fit. The strong predictive power of the model once again proves the effectiveness and robustness of the extended TPB model in explaining Generation Z social media behavior.

## Data Availability

The original contributions presented in the study are included in the article/Supplementary material, further inquiries can be directed to the corresponding author/s.
